# MicroRNA-574-5p promotes metastasis of non-small cell lung cancer by targeting PTPRU

**DOI:** 10.1038/srep35714

**Published:** 2016-10-20

**Authors:** Rui Zhou, Xiaoshu Zhou, Zhongyuan Yin, Jing Guo, Ting Hu, Shun Jiang, Li Liu, Xiaorong Dong, Sheng Zhang, Gang Wu

**Affiliations:** 1 Cancer Center, Union Hospital, Tongji Medical College, Huazhong University of Science and Technology, China; 2 Department of Oncology of the Affiliated Hospital of Qingdao University, Qingdao, China; 3 Department of Oncology, Second Xiangya Hospital of Central South University, Changsha, China

## Abstract

Dysregulation of microRNAs (miRNAs) has been associated with malignant behavior in a variety of cancers. Our previous study demonstrated that miRNA expression profiles are predictors for patients with advanced non-small cell lung cancer (NSCLC). We also showed that miRNAs are involved in small-cell lung cancer metastasis. Here, we used qRT-PCR to re-analyze our previous microarray results using serum samples from 75 patients with NSCLC. Surprisingly, we found that miR-574-5p and miR-874 were overexpressed in patients with metastatic advanced NSCLC but not in patients with non-metastatic advanced NSCLC. Additionally, miR-574-5p expression was correlated between matched serum and tissue samples from 68 patients. However, these 2 miRNAs are not prognostic factors for NSCLC. Transwell and wound-healing assays showed that miR-574-5p promotes the migration and invasion of NSCLC cells. Furthermore, miR-574-5p enhanced the tyrosine phosphorylation of β-catenin by repressing PTPRU expression *in vitro*. In conclusion, this study explored the expression of miR-574-5p in clinical samples and its molecular mechanisms in the metastasis of advanced NSCLC.

Lung cancer is the leading cause of cancer-related death in the world and is characterized by frequent mutations, amplifications, and epigenetic changes in different cancer-related genes^1^. Non-small cell lung cancer (NSCLC) accounts for the majority of lung cancers. Surgical resection remains the major treatment option for early-stage NSCLC; however, few patients can undergo curative resection. Comprehensive medical treatment is the standard therapy for advanced NSCLC. While advances in immunotherapy and targeted therapy are promising, the 5-year survival rate of patients with advanced lung cancer remains below 10%^1^. Almost all patients relapse, resulting in metastatic disease and death. Therefore, identification of new molecular targets that regulate the metastasis of NSCLC is urgently required.

Tyrosine phosphorylation is a molecular switch that is dynamically regulated by protein tyrosine phosphatases (PTPs) and kinases (PTKs). This molecular mechanism is involved in various cellular signaling processes that affect growth, differentiation, adhesion, apoptosis, migration, and invasion. Protein tyrosine phosphatase receptor type U (PTPRU), also known as Purkinje cell protein 2 (PCP2), is a PTP that has been shown to negatively regulate the growth and migration of colon cancer cells^2^. β-catenin plays an important role in cell adhesion and invasion, and it is a classical regulator of cancer. β-catenin can simultaneously stimulate the Wnt and E-cadherin pathways, and the interaction of microRNAs (miRNAs) and the β-catenin pathway has been widely investigated in different cancers^3^.

miRNAs are a class of non-coding endogenous RNA molecules that regulate gene expression at the post-transcriptional level. They suppress the translation of protein-coding genes by destabilizing or cleaving target mRNAs^4^. miRNAs play an important role in cancer development and are often associated with cancer prognosis^5^,^6^. In our previous study, we found that serum miRNA expression combinations are predictors of stage III/IV NSCLC^7^. A recent study demonstrated that miR-574-5p is a serum biomarker for early-stage NSCLC^8^. Additionally, our previous study showed that miR-574-5p promotes the invasion of small-cell lung cancer (SCLC) by regulating β-catenin signaling^9^. These results revealed a novel and important mechanism for regulating miR-574-5p and NSCLC metastasis.

The aim of this study was to identify the clinical importance and biological function of miR-574-5p in advanced-stage NSCLC. Assessment of miR-574-5p may be a minimally invasive approach to identify patients with metastatic (M) NSCLC, and miR-574-5p may be a novel therapeutic target for NSCLC.

## Results

### miR-574-5p and miR-874 overexpression in NSCLC patients’ serum samples

Serum samples from 75 patients with NSCLC [30 with non-metastatic (NM) NSCLC and 45 with M NSCLC] were included in this study (Table 1). For 68 patients, both serum and *in situ* tumor tissue samples were included. There were no significant differences in the distribution of age, gender, smoking status, Eastern Cooperative Oncology Group (ECOG) status, T stage, N stage or histological classification between patients with NM and M NSCLC.

**Figure 1:**
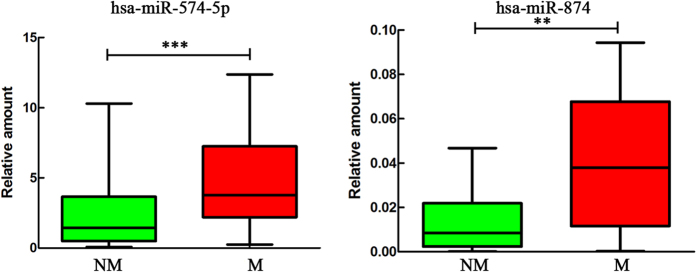
qRT-PCR validation of miR-574-5p and miR-874 overexpression in metastatic NSCLC patient serum samples. The results were analyzed using Mann-Whitney U tests. *p < 0.05; **p < 0.01; ***p < 0.001. Red, metastatic group; green, non-metastatic group.

We selected 7 miRNA candidates (miR-184, miR-574-5p, miR-874, miR-3074-5p, miR-4459, miR-4746-3p and miR-4685-5p) and assessed their relationships to SCLC stage in our previous study^9^. In this study, we determined the relative abundance of these miRNAs, and all of them yielded acceptable and consistent signals (data not shown). We next performed qRT-PCR assays to measure the expression levels of these miRNAs in serum samples, and Mann-Whitney U tests were performed. Of these 7 miRNAs, miR-574-5p (p < 0.001) and miR-874 (p = 0.002) were significantly overexpressed in the M NSCLC samples (Table 2). A boxplot diagram shows the relationship between these 2 miRNAs and metastasis (Fig. 1).

### miR-574-5p expressions in matching tissue and serum samples show a linear correlation

**Figure 2:**
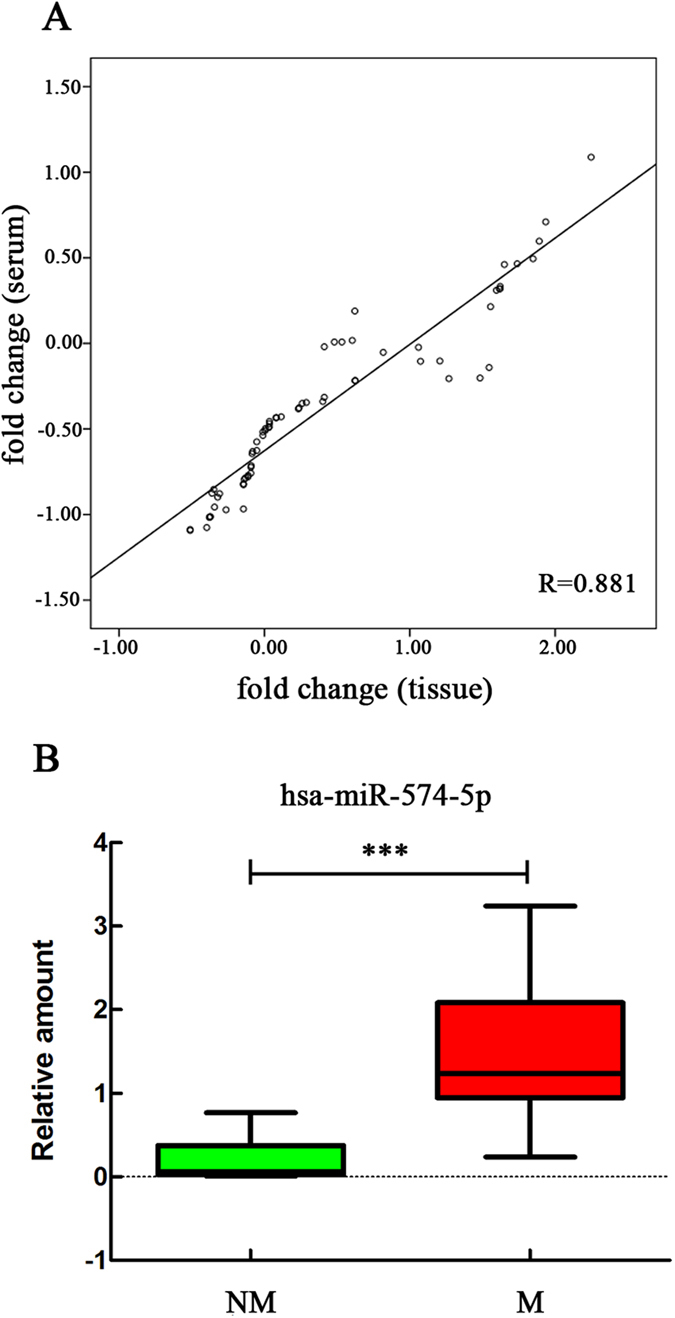
miR-574-5p expression in NSCLC tissue samples. (**A**) Pearson correlation scatter plot of miR-574-5p expression in matched NSCLC patients’ tissue and serum samples. (**B**) qRT-PCR of miR-574-5p overexpression in metastatic NSCLC tissue samples as analyzed using the Mann-Whitney U test. *p < 0.05; **p < 0.01; ***p < 0.001.

To assess the correlation between the miRNA levels in tissue and serum samples, we investigated the expressions of miR-574-5p and miR-874 in 45 matching tissue and serum samples (Supplementary Table 1). The results showed that the miR-574-5p expression profiles were significantly correlated (p < 0.001; r = 0.881; Fig. 2A), which suggested that serum levels of this miRNA could reflect tissue expression. Consequently, we compared the miRNA levels between the tissues from the M and NM groups. The results showed that miR-574-5p (p < 0.001) was significantly associated with metastasis. A boxplot diagram shows the differential expression between NM and M NSCLC patients (Fig. 2B).

### miR-574-5p and miR-874 are not predictors of progression-free survival/overall survival (PFS/OS) in NSCLC

**Figure 3:**
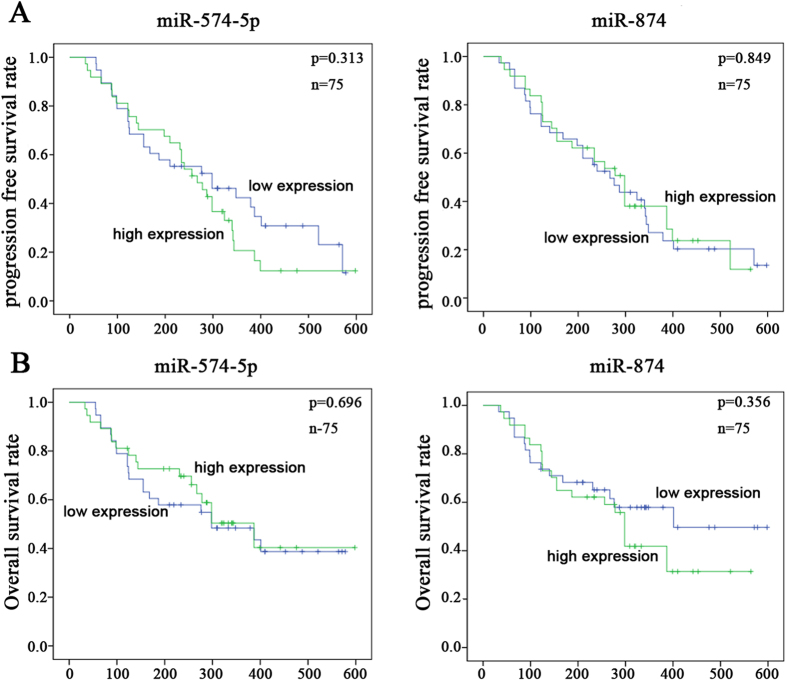
Correlation between the expression of miR-574-5p/miR-874 and prognosis in patients with NSCLC. (**A**) K-M analysis indicated that miR-574-5p and miR-874 were not associated with PFS. (**B**) K-M analysis indicated that miR-574-5p and miR-874 were not associated with OS.

To determine whether miR-574-5p and miR-874 are associated with PFS and OS of patients with NSCLC, we investigated the same 75 NSCLC patients. The median follow-up time for these patients was 346 days. During the follow-up period, 55 patients (73.3%) exhibited disease progression, 15 patients (20%) were lost to follow-up, and 38 (50.6%) died due to NSCLC. We next performed a Kaplan-Meier (K-M) analysis (log-rank test) to determine whether miR-574-5p and miR-874 were predictive factors for advanced NSCLC. The K-M analysis revealed that miR-574-5p and miR-874 were not prognostic factors for PFS or OS (Fig. 3). Thus, neither serum miR-574-5p nor miR-874 expression was correlated with the prognosis of advanced NSCLC.

### miR-574-5p and miR-874 transfection in the A549, H1975 and PC9 NSCLC cell lines

In the present study, A549, H1975 and PC9 cells were chosen to represent typical NSCLC cell lines. To investigate the biological effects of miR-574-5p and miR-874 on the invasiveness of NSCLC, we performed *in vitro* functional analyses using overexpression and inhibition strategies based on miRNA mimics and inhibitors, respectively, which were transfected into A549, H1975 and PC9 cells. The qRT-PCR results indicated that the miRNAs were substantially overexpressed or significantly inhibited after 72 h of treatment with the transfection mimics and inhibitors, respectively (Supplementary Figure S1).

### miR-574-5p promotes NSCLC migration and invasion *in vitro*, whereas miR-874 has no effect

**Figure 4:**
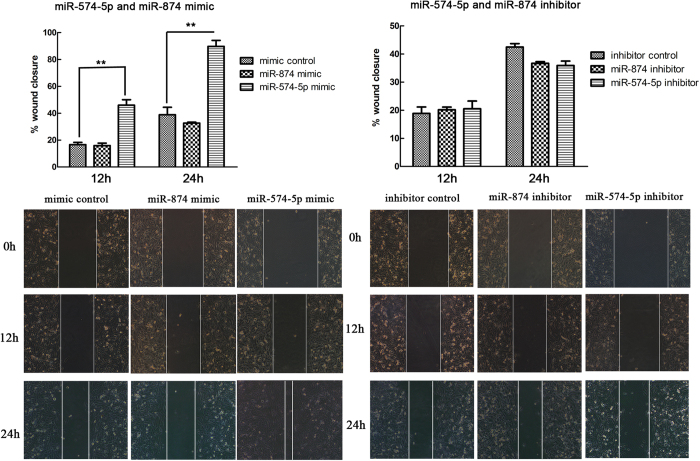
miR-574-5p promoted the wound-healing process in A549 cells, whereas miR-874 did not contribute to wound healing. Upper panel, wound-healing results for A549 cells transfected with the indicated mimics or inhibitors (analyzed by t-tests). Lower panel, images of wounds of A549 cells transfected with the indicated mimics or inhibitors taken 0, 12 and 24 h after the wounds were inflicted (×40). *p < 0.05; **p < 0.01; ***p < 0.001.

**Figure 5:**
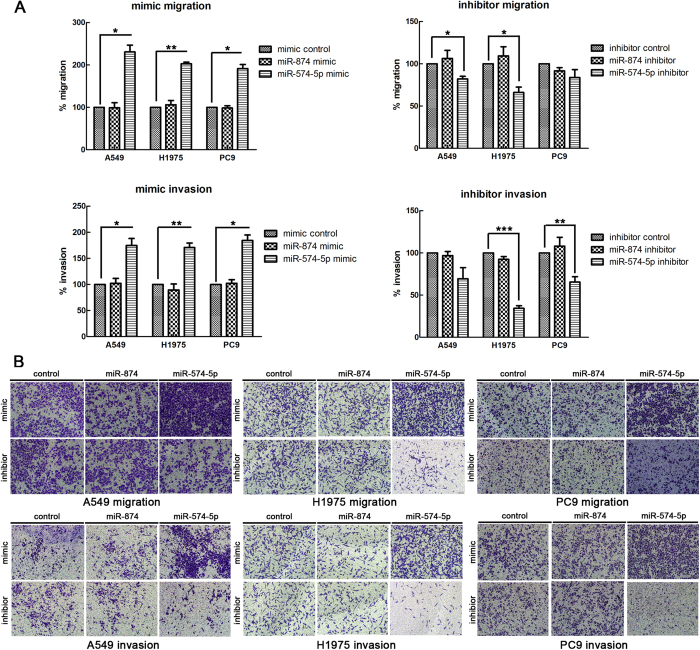
miR-574-5p promotes the metastasis and invasion of NSCLC cell lines, whereas miR-874 did not contribute to these processes. (**A**) Upper panel, statistical results for the Transwell migration assays of A549, H1975 and PC9 cells transfected with the indicated mimics or inhibitors (analyzed by t-tests). Lower panel, statistical results for the Transwell invasion assays of A549, H1975 and PC9 cells transfected with the indicated mimics or inhibitors (analyzed by t-test); % migration = [mean number of cells invading the membrane/mean number of cells migrating through the control insert membrane] × 100; % invasion = [mean number of cells invading the matrigel membrane/mean number of cells migrating through the control insert membrane] × 100. (**B**) Upper panel, images from the Transwell migration assays of A549, H1975 and PC9 cells (×100). Lower panel, images from the Transwell invasion assays of A549, H1975 and PC9 (×100). *p < 0.05; **p < 0.01; ***p < 0.001.

The above clinical findings suggested that miR-574-5p and miR-874 may participate in NSCLC progression. Cell proliferation, migration and invasion are commonly required for metastatic progression. We first examined the effects of miR-574-5p and miR-874 on proliferation of A549, H1975 and PC9 cells using MTT assays and found that none of them affected cell growth (Supplementary Figure S2). Next, to evaluate whether these miRNAs actually increase the metastatic ability of NSCLC, we conducted wound-healing assays, which indicated that miR-574-5p (12 h, p = 0.003; 24 h, p = 0.002) significantly enhanced the migration of A549 cells. However, miR-874 did not affect cell migration (Fig. 4). Furthermore, Transwell assays were used to verify these findings. As expected, migration and matrigel invasion assays both confirmed that miR-574-5p promoted metastasis of the NSCLC cell lines, whereas miR-874 did not (Fig. 5).

### miR-574-5p represses PTPRU and thus increases β-catenin tyrosine phosphorylation in NSCLC

**Figure 6:**
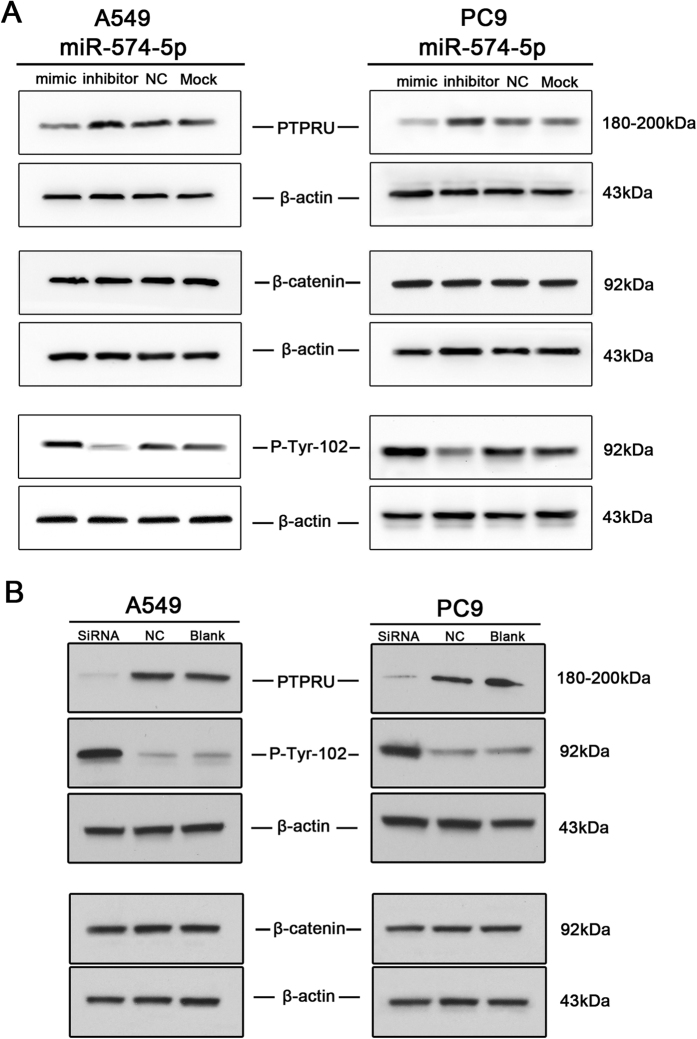
miR-574-5p overexpression inhibited PTPRU and enhanced the tyrosine phosphorylation of β-catenin in A549 and PC9 cells as determined by immunoblot analysis. (**A**) immunoblot of PTPRU, β-catenin and the P-Tyr-102 of β-catenin in A549 and PC9 cells after transfection with miR-574-5p mimic or inhibitor compared with control group. (**B**) immunoblot of PTPRU, β-catenin and the P-Tyr-102 of β-catenin in A549 and PC9 cells after transfection with SiRNA of PTPRU compared with control group.

As we reported previously, at least 2 different sites of the 3′ untranslated region (3′ UTR) of the PTPRU sequence are complementary to miR-574-5p, which thus represses PTPRU protein expression in SCLC cell lines^9^. Consequently, we further investigated the expression of PTPRU in miR-574-5p-overexpressing NSCLC cells and obtained similar results (Fig. 6). These data confirmed that PTPRU was a functional target of miR-574-5p in NSCLC, and this miRNA may enhance the migration and invasion of NSCLC by repressing PTPRU expression.

PTPRU has been reported^2^,^10^ to inhibit cell growth and invasion by decreasing β-catenin tyrosine phosphorylation. Because the presence of phosphorylated tyrosine residues on β-catenin correlates with a loss of E-cadherin-mediated cell adhesion, PTPRU-mediated dephosphorylation of β-catenin contributes to enhanced cell adhesion^2^. Our previous study demonstrated that transfection with miR-574-5p enhanced the tyrosine phosphorylation of β-catenin in SCLC cell lines^9^. Thus, western blot analysis was used to examine the tyrosine phosphorylation of β-catenin in miR-574-5p-overexpressing NSCLC cell lines. As shown in Fig. 6, transfection with miR-574-5p enhanced the tyrosine phosphorylation of β-catenin, and its inhibition led to decreased phosphorylation. To determine whether decreased PTPRU actually enhance the β-catenin tyrosine phosphorylation in NSCLC, we used SiRNA of PTPRU to repress it in cell lines. The results revealed that repression PTPRU can significantly promote the tyrosine phosphorylation of β-catenin (Fig. 6). Consequently, miR-574-5p overexpression can activate the β-catenin signaling pathway. Repressing miR-574-5p expression may contribute to enhanced cell adhesion and reduced tumor metastasis by reducing β-catenin tyrosine phosphorylation.

Based on these experimental studies and clinical data, we concluded that miR-574-5p promotes NSCLC metastasis and is a potential therapeutic target for the prevention of metastasis in patients with NSCLC. Additionally, the tyrosine phosphorylation of β-catenin plays an important role in this process.

## Discussion

During the past few decades, precise treatment of NSCLC has developed rapidly; however, NSCLC remains the leading cause of cancer-related death worldwide^1^. With the widespread use of PET/CT, systemic micro-metastasis nodules can now be detected. However, this detection does not replace pathological diagnosis, and some metastases cannot be identified due to insufficient metabolic changes^11^. Patients with advanced NSCLC who receive incomplete resection may develop abnormal activation of the immune system, causing accelerated tumor growth. This process may be responsible for early recurrence in some patients with early-stage NSCLC. However, pathologically diagnosing every suspicious nodule is currently impossible. Thus, a noninvasive and easy sampling strategy that provides reliable information on the metastatic state of NSCLC is urgently required.

Despite the importance of staging, almost all patients with NSCLC eventually relapse, resulting in metastatic disease and death^12^. lthough new advances in immunotherapy and targeted therapy are promising, once systemic metastasis develops, the 5-year survival rate is below 10%^1^. Thus, inhibiting metastatic progression to prolong the OS of patients with NSCLC remains a challenge.

In conclusion, an easily accessible and detectable molecule is urgently needed as a biomarker of clinical stage as well as a potential therapeutic target. Circulating miRNAs are promising as they have been reported to have potential roles in cancer diagnosis and treatment. Although plasma has high RNase activity^13^, accumulating evidence has indicated that endogenous circulating miRNAs are resistant to plasma RNase activity^14^. In fact, miRNA profiles in the blood could reflect the primary tumor and may be detectable during the early stages of tumor development; thus, miRNA profiles could be useful for obtaining real-time information on the tumor status.

Foss *et al.* reported that miR-574-5p was differentially expressed between the control and stage I to stage II NSCLC patient samples^8^. Additionally, miR-574-5p has been shown to be differentially expressed in NSCLC in other studies^15^,^16^. Li *et al.* found that miR-574-5p enhanced the tumor progression of NSCLC cells *in vivo*, indicating that miR-574-5p may be involved in the metastasis of human lung cancer cells^17^. It also has been reported to be an oncogene in various types of cancer. This miRNA has been shown to be up-regulated in esophageal squamous cell carcinoma and colorectal cancer tumor tissues compared with adjacent non-tumor tissues, and could promote cell growth of thyroid cancer cells^5^,^18^,^19^. While miR-574-5p has been widely explored as an oncogene, miR-874 has been shown to act as a tumor suppressor. miR-874 has been reported to inhibit cell proliferation, invasion and angiogenesis in gastric cancer^20^,^21^. Nohata *et al.* claimed that miR-874 inhibits cell proliferation and maxillary sinus squamous cell carcinoma (MSSCC) invasion by directly regulating PPP1CA^22^.

Here, we sought to identify miRNAs related to NSCLC metastasis. Because our previous study identified several circulating miRNAs related to SCLC metastasis, we re-confirmed the expression of these miRNAs in serum samples from patients with NSCLC. We selected miR-574-5p and miR-874 because they were overexpressed in M NSCLC patient serum samples, as detected by qRT-PCR, in a cohort of 75 NSCLC patients. Additionally, serum miR-574-5p expression had a strong positive correlation with that in primary tumor samples. Therefore, miR-574-5p overexpression in patients with NSCLC is strongly suggestive of the metastatic state. These patients should be carefully and comprehensively assessed before surgery to determine whether they require parallel adjuvant therapy. Kesanakurti *et al.* reported that miR-874 expression is reduced in NSCLC tissue specimens and that its up-regulation leads to the inhibition of NSCLC cell invasion^23^. However, we did not find the same results, potentially due to the different clinical samples and different cell lines studied. Additional studies will be needed to further explore these results.

There are hundreds of published studies proposing the value of different miRNAs as prognostic biomarkers in every different type of cancer. Ling *et al.* reported that miR-224 is a negative prognostic factor for patients with colorectal cancer^24^. A large study in patients with B cell lymphoma found that high expression levels of miR-155 were significantly associated with rituximab plus cyclophosphamide, doxorubicin, vincristine and prednisone (R-CHOP) treatment failure^25^. In our previous study, Jing used a training set to develop a prognostic panel of miR-1, miR-30d, miR-221, and miR-486 in advanced NSCLC, and this panel was validated in a larger independent set of samples^7^.

This study attempted to estimate the prognostic values of serum miR-574-5p and miR-874. Unfortunately, neither miR-574-5p nor miR-874 had prognostic value for patients with NSCLC in this study, although our previous research indicated that miR-574-5p was a prognostic risk factor for SCLC^9^. Nevertheless, the histological origin, clinical manifestation, therapeutic response and prognosis of SCLC and NSCLC vary enormously; hence, the same oncogene may perform various roles in different cancers. Additionally, our study included a small number of participants, approximately 20% of which were lost to follow up, and only half of the patients died. Thus, the prognostic roles of miR-574-5p and miR-874 should be assessed in a larger population. It has been widely reported that knockdown of PTPRU suppresses growth and motility and β-catenin transcriptional activity of gastric cancer and glioma cells^26^,^27^. However, up to now, there is no report about the relationship between PTPRU and cancer prognosis. In this present study, PTPRU acts as the functional target of miR-574-5p. The prognostic role of it remains unclear and need to be further investigated.

For metastasis, cancer cells must intravasate into a blood vessel or lymph vessel from the primary tumor and then extravasate out of the vessel at a distant metastatic site. This complicated process is linked to different biological behaviors and signaling pathways. Numerous *in vitro* and *in vivo* studies have demonstrated the ability of miRNAs to inhibit all hallmarks of cancer, ultimately resulting in tumor regression. Recently, a study claimed that miR-21 promotes the expression of downstream VEGF and thus serves as a regulator of angiogenesis, which is involved in the cancer invasion process^28^. Lin *et al.* demonstrated that miR-135b promotes lung cancer metastasis by regulating multiple targets in the Hippo pathway and LZTS1^29^. Our present study showed that miR-574-5p could promote the migration and invasion of NSCLC cell lines *in vitro*. And down-regulation it could repress the ability. Additionally, miR-574-5p overexpression led to the down-regulation of PTPRU, thus repressing the PTPRU-mediated dephosphorylation of β-catenin, which contributes to enhanced cell adhesion in NSCLC. Therefore, miR-574-5p may promote NSCLC metastasis by ultimately targeting PTPRU and increasing the phosphorylation of β-catenin.

In conclusion, our study demonstrated that miR-574-5p and miR-874 could distinguish patients with M NSCLC from those with NM NSCLC and that the serum and tissue expressions of these miRNAs were correlated. However, these 2 miRNAs are not prognostic factors of NSCLC. In this study, we found that miR-574-5p promotes the metastatic ability of NSCLC cell lines, while miR-874 does not. miR-574-5p may participate in the metastatic process by targeting PTPRU and enhancing the tyrosine phosphorylation of β-catenin; thus, miR-574-5p might be a new target for NSCLC therapy.

## Materials and Methods

### Patients and clinical samples

Serum and tumor biopsy samples from 75 NSCLC patients were used in this study. The experiments were approved by the Ethics Review Board of Wuhan Union Hospital, Tongji Medical College, Huazhong University of Science and Technology and carried out in accordance with the ethical guidelines of this institution. All patients were informed and provided both written and oral consent before the samples were collected. These patients had an ECOG performance status of 0–1 from the Wuhan Union Hospital between November 2012 and May 2013. None of the patients had undergone treatment before enrollment. All participants underwent strict imaging and physical examinations, and their histories, including demographic characteristics and medical and smoking histories, were recorded. All of the enrolled patients were diagnosed by pathology and staged by specialized oncologists via the 7^th^ edition of the International Association for the Study of Lung Cancer TNM Classification. After patients were admitted to the hospital, a 5 ml peripheral blood sample was drawn into a gold-top serum-separating tube, processed for serum extraction within 2 h, and then incubated at −80 °C for long-term storage. In total, 68 patients had their tumor tissues biopsied by CT-guided percutaneous lung biopsy, which yielded approximately 1 × 0.2 cm biopsy samples that were then incubated in 1.5 ml of RNAlater solution (Ambion, AM7021) and subsequently stored at −80 °C. Serum and tissue samples were selected retrospectively at the time of analysis according to the following requirements: 1. the patient had been diagnosed with NSCLC; 2. a sufficient volume of serum was available for RNA isolation; and 3. demographic, clinical, and follow-up data were available for the patient. All patients received the standard treatments recommended by the National Comprehensive Cancer Network (NCCN) guidelines for NSCLC.

### Total RNA extraction

Total RNA was isolated from 400 μl serum samples using a mirVana^TM^ PARIS^TM^ Kit (Applied Biosystems, AM1556) following the manufacturer’s protocol. A total of 25 fmol synthetic *C. elegans* miRNA (cel-miR-39-3p, Qiagen) was introduced after the addition of denaturing solution to each sample to monitor technical variations in RNA extraction as previously described^30^. Total RNA was isolated from cells and tissues using an E.Z.N.A.^TM^ Total RNA Kit II (OMEGA R6934-02) following the manufacturer’s protocol.

### Reverse transcription PCR and qRT-PCR

Each miRNA was specifically reverse transcribed according to the manufacturer’s protocol using a TaqMan MicroRNA Reverse Transcription Kit with a stem-loop RT primer (Applied Biosystems, 4366596). For real-time PCR, 2 μl of diluted reverse transcription product was mixed with 10 μl of SYBR Select Master Mix (2×), 0.8 μl of forward and reverse primers and 6.4 μl of nuclease-free water to a final volume of 20 μl (Applied Biosystems, SYBR Select Master Mix, 4472908). All reactions were performed in triplicate on a StepOnePlus Real-Time PCR System (Applied Biosystems) under the following conditions: 50 °C for 2 min, 95 °C for 2 min, followed by 40 cycles at 95 °C for 3 s and 60 °C for 30 s. The TaqMan stem-loop primer for reverse transcription PCR and the forward and reverse primers for real-time PCR are shown in Supplemental Table 1.

### Cells and cell culture

The human NSCLC cell lines A549, H1975 and PC9 were purchased from the Cell Resource Center, Shanghai Institute of Biochemistry and Cell Biology, Chinese Academy of Sciences. The cells were maintained at 37 °C in a humidified air atmosphere containing 5% carbon dioxide and cultured in RPMI 1640 (HyClone, SH30809.01B) containing 10% heat-inactivated fetal bovine serum (Gibco, 16000-044).

### Mimics/inhibitors/SiRNA for transfection

All mimics, inhibitors and SiRNA were designed and constructed by RiboBio (Guangdong, China). Cells were transfected with mimics, inhibitors or SiRNA using Lipofectamine 2000 (Invitrogen, 11668-019) following the manufacturer’s protocol. The final concentration of the mimics was 50 nM and that of the inhibitors/SiRNA was 100 nM. The cells were subjected to further experimentation at least 48 h after transfection. The transfection efficiencies of all mimics and inhibitors were verified (Supplemental Fig. 1).

### MTT assays

For the MTT assays, 4000 cells/well were plated in 96-well plates and transfected with mimics or inhibitors for 24 h. After transfection for 24, 48 and 72 h, 20 μl of 5% MTT (Sigma, M5655) was added to each well, and the culture was continued for 4 h and then terminated with 200 μl of DMSO (Sigma, D4540). Absorbances at 490 nm were measured using a Bio-Tek M450 instrument.

### Wound-healing assays

Approximately 5 × 10^5^ A549 cells were suspended in 2 ml of complete medium, plated in 6-well plates and cultured at 37 °C for approximately 24 h. Once cell monolayers had formed, wounds were generated by scraping with a 200 μl plastic pipette tip. The monolayers were rinsed several times with medium to remove the dislodged cells, and the culture was continued in 2% serum at 37 °C for 12 or 24 h in an incubator containing 5% CO_2_. Cells that had migrated into the wound area were imaged using an inverted microscope at 100x magnification (Olympus, CKX41). The distance was measured using ImageJ2x.

### *In vitro* migration and invasion assays

For the Transwell migration assays, 2 × 10^4^/200 μl of A549, H1975 or PC9 cells was plated in the top chamber with a non-coated membrane (24-well insert, pore size: 8 μm, BD Biosciences). For the invasion assays, 5 × 10^4^/200 μl of A549, H1975 or PC9 cells was plated in the top chamber with extracellular matrix gel (Sigma, E1270)-coated membranes (24-well insert, pore size: 8 μm, BD Biosciences). In both assays, the cells were plated in medium without serum, and medium with 15% serum was used as a chemoattractant in the lower chamber. The cells were incubated at 37 °C for 24 h, and cells that did not migrate through or invade the pores were removed with a cotton swab. Cells on the lower surface of the membrane were fixed with methanol and stained with 0.1% crystal violet (Sigma). Cells in 9 random fields of view at 100x magnification were counted and expressed as the average number of cells per field of view. Images were acquired using an inverted phase-contrast microscope at 100x magnification (Olympus, CKX41).

### Western blot analysis

Western blot analysis was performed according to standard procedures as previously described^31^. The following primary antibodies were used: anti-PTPRU mAb (1:1000; R&D Systems^®^, MAB7475), anti-P-Tyr-102 mAb (1:2000; CST, 9416S), and anti-β-actin mAb (1:2000; Santa Cruz, sc-47778). The following secondary antibodies were used: peroxidase-conjugated Affinipure goat anti-mouse IgG (H+L) (1:3000; Proteintech, SA00001-1) and peroxidase-conjugated Affinipure goat anti-rabbit IgG (H+L) (1:3000; Proteintech, SA00001-2).

### Statistical analysis

All statistical analyses were conducted using SPSS 21.0 statistical software. The Mann-Whitney U test was used to determine the significance of differences between the miRNA levels in the serum or tissue of patients with NM and M NSCLC. The correlation between miRNA expression in the serum and tissue was determined using Pearson correlation coefficients in a two-tailed test. The log-rank test (based on K-M analysis) and Cox proportional hazards regression were used to analyze the effect of the clinical variables and miRNAs on the patients’ PFS and OS. Continuous data were compared using Student’s 2-tailed t-test. In all cases, p < 0.05 was considered statistically significant.

## Additional Information

**How to cite this article**: Zhou, R. *et al.* MicroRNA-574-5p promotes metastasis of non-small cell lung cancer by targeting PTPRU. *Sci. Rep.* **6**, 35714; doi: 10.1038/srep35714 (2016).

## Supplementary Material

Supplementary Information

## Figures and Tables

**Table 1:** Selected characteristics of the NSCLC patient population recruited at the cancer center of Wuhan Union Hospital between 2012-11-28 and 2013-5-28.

Viable	Non-metastasis	Metastasis	p-value
	(N = 30)	(N = 45)	(NM vs. M)
Age^a^, years			
Mean/Median	59.1/60.5	60.3/61	0.918
Range	44-73	38-80	
Gender^b^, *n* (%)			
Male	20 (66.7)	27 (60.0)	0.559
Female	10 (33.3)	18 (40.0)	
Smoking status^b^, *n* (%)			
Never	6 (20.0)	5 (11.1)	0.211
Former*	10 (33.3)	24 (53.3)	
Current	14 (46.7)	16 (35.6)	
ECOG status^b^, *n* (%)			
0	16 (50.3)	24 (53.3)	1.000
1	14 (46.7)	21 (46.7)	
T stage^b^, *n* (%)			
T1/2	10 (33.3)	19 (42.2)	0.439
T3/4	20 (66.7)	26 (57.8)	
N stage^b^, *n* (%)			
N0/1	8 (26.7)	11 (24.4)	0.828
N2/3	22 (73.4)	34 (75.6)	
Histological classification^b^, *n* (%)			
Adenocarcinoma	24 (80.0)	34 (75.6)	0.652
SCC	6 (20.0)	11 (24.4)	
MFT, days (range)	346 (120–598)		

^a^Used t-test.

^b^Used chi-square test.

^*^Former smokers were subjects who quit smoking 6 months or more before the study.

Abbreviations: M, metastasis; NM, non-metastasis; SCC, squamous cell carcinoma; MFT, median follow-up time. The follow-up time ended on 2015-12-22.

**Table 2:** Serum miRNAs significantly correlated with NSCLC metastasis.

No. samples	Mean Rank		Mann-Whitney U test
	30	45	
miRNA name	NM	M	Sig.
hsa-mir-184	36.33	39.11	0.589
**hsa-mir-574-5p**	30.77	48.82	<0.001***
**hsa-mir-874**	33.43	47.71	0.002**
hsa-mir-3074-5p	38.08	37.94	0.978
hsa-mir-4459	36.17	39.22	0.552
hsa-mir-4756-3p	42.48	35.01	0.146
hsa-mir-4685-5p	35.32	39.79	0.384

NOTE: Bold indicates significantly different expression between the NM and M groups.

Abbreviations: NM, non-metastasis; M, metastasis. *p-value < 0.05; **p-value < 0.01; ***p-value < 0.001.
