# Supplementary material for: MicroRNA-574-5p promotes metastasis of non-small cell lung cancer by targeting PTPRU
Source: Sci Rep. 2016 Oct 20;6:35714. doi: 10.1038/srep35714 (PMC5071770; doi:10.1038/srep35714)
Supplement: Supplementary Information [file srep35714-s1.doc]

**Title:** MicroRNA-574-5p promotes metastasis of non-small cell lung cancer by targeting PTPRU

**Authors:** Rui Zhou^1^, Xiaoshu Zhou^1^, Zhongyuan Yin^1^, Jing Guo^2^, Ting Hu^1^, Shun Jiang^3^, Li Liu^1^, Xiaorong Dong^1^, Sheng Zhang^1^, Gang Wu^1^

**Affiliations:**

^1^Cancer Center, Union Hospital, Tongji Medical College, Huazhong University of Science and Technology, China

^2^Department of Oncology of the Affiliated Hospital of Qingdao University, Qingdao, China

^3^Department of Oncology, Second Xiangya Hospital of Central South University, Changsha, China

**Corresponding author:**

**Full name:** Prof. Gang Wu, MD

**E-mail address:** [xhzlwg@163.com](mailto:xhzlwg@163.com)

**Mailing address:** Cancer Center, Union Hospital, Tongji Medical College, Huazhong University of Science and Technology, 1277 JieFang Avenue, Wuhan 430022, China

**Telephone:** +86 138 7124 0042

**Fax:** +86 02765650733

| Viable | | Non-metastasis | Metastasis | p-value |
| --- | --- | --- | --- | --- |
|  | | (N=28) | (N=40) |  |
| Age^a^, years | |  |  |  |
|  | Mean/Median | 58.8(59) | 59.8(59.5) | 0.998 |
|  | Range | 44-77 | 38-75 |  |
| Gender^b^, *n*(%) | |  |  |  |
|  | Male | 18(64.3) | 24(60.0) | 0.720 |
|  | Female | 10(35.7) | 16(40.0) |  |
| Smoking status^b^, *n*(%) | |  |  |  |
|  | Never | 6(21.4) | 5(12.5) | 0.351 |
|  | Former | 10(35.7) | 21(52.5) |  |
|  | Current | 12(42.9) | 14(35.0) |  |
| ECOG status^b^, *n*(%) | |  |  |  |
|  | 0 | 14(50.0) | 20(50.0) | 1.000 |
|  | 1 | 14(50.0) | 20(50.0) |  |
| T stage^b^, *n*(%) | |  |  |  |
|  | T1/2 | 9(32.1) | 22(55.0) | 0.305 |
|  | T3/4 | 19(67.9) | 28(45.0) |  |
| N stage^b^, *n*(%) | |  |  |  |
|  | N0/1 | 9(32.1) | 11(27.5) | 0.679 |
|  | N2/3 | 19(67.9) | 29(72.5) |  |
| Histological classification^b^, *n*(%) | |  |  |  |
|  | Adenocarcinoma | 23(82.1) | 30(75.0) | 0.484 |
|  | SCC | 5(17.9) | 10(25.0) |  |

Supplemental Table 1. Selected characteristics of the SCLC patients from whom both serum and tissue were collected.

a Used t-test

b Used Chi-square test

Abbreviations: SCC, squamous cell carcinoma; MFT, median follow-up time.

| cel-miR-39-3p | Sequence | UCACCGGUGUAAAUCAGCUUG | |
| --- | --- | --- | --- |
|  | TaqMan primer | GTCGTATCCAGTGCAGGGTCCGAGGTATTCGCACTGGACGACCAAGCT | |
|  | PCR-F | TCCGTCTCCGGGTGTAAATCAG | |
|  | PCR-R | CAGTGCAGGGTCCGAGGTAT | |
| hsa-miR-4685-5p | Sequence | CCCAGGGCUUGGAGUGGGGCAAGGUU |  |
|  | TaqMan primer | GTCGTATCCAGTGCAGGGTCCGAGGTATTCGCACTGGATACGACAACCT |  |
|  | PCR-F | TTACCCAGGGCTTGGAGTG |  |
|  | PCR-R | CAGTGCAGGGTCCGAGGTAT |  |
| hsa-miR-4746-3p | Sequence | AGCGGUGCUCCUGCGGGCCGA |  |
|  | TaqMan primer | GTCGTATCCAGTGCAGGGTCCGAGGTATTCGCACTGGATACGACTCGGCC |  |
|  | PCR-F | ATTAAGCGGTGCTCCTGCG |  |
|  | PCR-R | CAGTGCAGGGTCCGAGGTAT |  |
| hsa-miR-3074-5p | Sequence | GUUCCUGCUGAACUGAGCCAG |  |
|  | TaqMan primer | GTCGTATCCAGTGCAGGGTCCGAGGTATTCGCACTGGATACGACCTGGCT |  |
|  | PCR-F | GCACGTTCCTGCTGAACTGA |  |
|  | PCR-R | CAGTGCAGGGTCCGAGGTAT |  |
| hsa-miR-874 | Sequence | CUGCCCUGGCCCGAGGGACCGA |  |
|  | TaqMan primer | GTCGTATCCAGTGCAGGGTCCGAGGTATTCGCACTGGATACGACTCGGTC |  |
|  | PCR-F | ATTACTGCCCTGGCCCGA |  |
|  | PCR-R | CAGTGCAGGGTCCGAGGTAT |  |
| hsa-miR-4459 | Sequence | CCAGGAGGCGGAGGAGGUGGAG |  |
|  | TaqMan primer | GTCGTATCCAGTGCAGGGTCCGAGGTATTCGCACTGGATACGACCTCCAC |  |
|  | PCR-F | TTAACCAGGAGGCGGAGGAG |  |
|  | PCR-R | CAGTGCAGGGTCCGAGGTAT |  |
| hsa-miR-184 | Sequence | UGGACGGAGAACUGAUAAGGGU |  |
|  | TaqMan primer | GTCGTATCCAGTGCAGGGTCCGAGGTATTCGCACTGGATACGACACCCTT |  |
|  | PCR-F | GCGTTGGACGGAGAACTGAT |  |
|  | PCR-R | CAGTGCAGGGTCCGAGGTAT |  |
| hsa-mir-574-5p | Sequence | UGAGUGUGUGUGUGUGAGUGUGU |  |

Supplemental Table 2. The TaqMan stem-loop primers for reverse transcription PCR and the forward and reverse primers for real-time PCR. *The primer sets for hsa-mir-574-5p (ssD809231223, MQP-0101) were purchased form RiboBio, Guangdong, China. The sequences of the primer sets for hsa-mir-574-5p belong to the same commercial company (RiboBio, Guangdong, China) and are not currently publicly available.


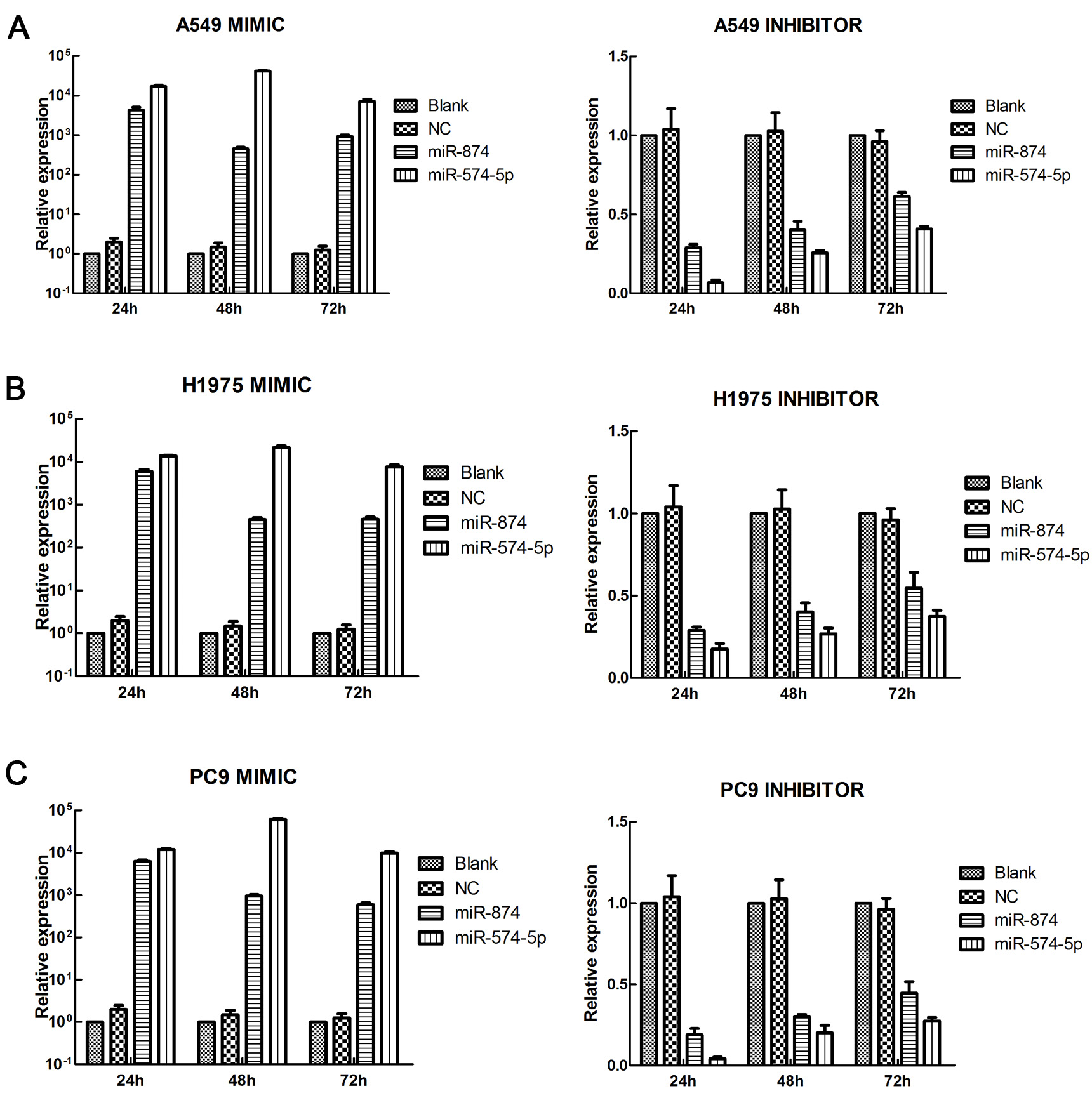


Supplementary Figure S1 miR-574-5p and miR-874 were markedly overexpressed or significantly inhibited after 72 hours of treatment with transfection mimics and inhibitors, respectively, of A549 (A), H1975 (B) and PC9 (C) cells compared with the NC and blank control groups, as validated by qRT-PCR.


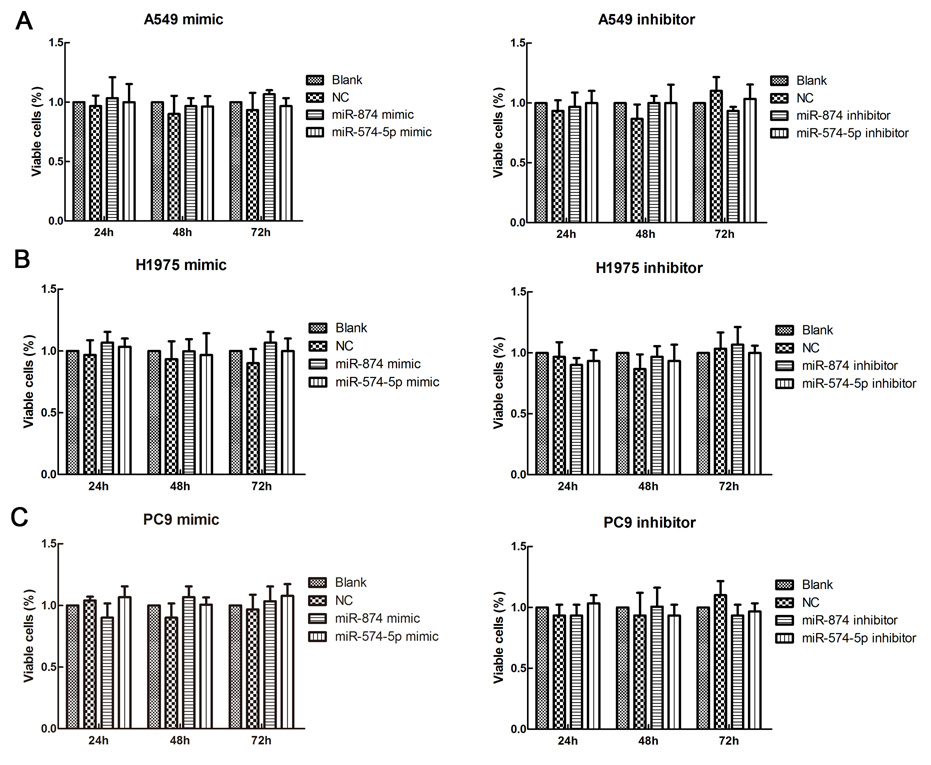


Supplementary Figure S2 miR-574-5p and miR-874 mimic/inhibitor did not affect cell growth of A549 (A), H1975 (B) and PC9 (C) cells compared with the NC and blank control groups, as validated by MTT assay.
